# PTPLAD1 Regulates PHB-Raf Interaction to Orchestrate Epithelial-Mesenchymal and Mitofusion-Fission Transitions in Colorectal Cancer

**DOI:** 10.7150/ijbs.82361

**Published:** 2024-03-25

**Authors:** Zi-Jia Huang, Yang-Jia Li, Jie Yang, Lei Huang, Qian Zhao, Yi-Fan Lu, Yang Hu, Wei-Xia Zhang, Jun-Ze Liang, Jinghua Pan, Yun-Long Pan, Qing-Yu He, Yang Wang

**Affiliations:** 1 MOE Key Laboratory of Tumor Molecular Biology and State Key Laboratory of Bioactive Molecules and Druggability Assessment, College of Life Science and Technology, Jinan University, Guangzhou 510632, China.; 2 Department of Molecular, Cell and Cancer Biology, Program in Molecular Medicine, University of Massachusetts Medical School, 364 Plantation Street, Worcester, MA, 01605, USA.; 3 Department of General Surgery, The First Affiliated Hospital of Jinan University, Guangzhou, China.

**Keywords:** PTPLAD1, PHB, Raf, ERK, Colorectal cancer, Metastasis, Epithelial-to-mesenchymal transition, Mitofission

## Abstract

Colorectal cancer (CRC) remains one of the leading causes of cancer-related death worldwide. The poor prognosis of this malignancy is attributed mainly to the persistent activation of cancer signaling for metastasis. Here, we showed that protein tyrosine phosphatase-like A domain containing 1 (PTPLAD1) is down-regulated in highly metastatic CRC cells and negatively associated with poor survival of CRC patients. Systematic analysis reveals that epithelial-to-mesenchymal transition (EMT) and mitochondrial fusion-to-fission (MFT) transition are two critical features for CRC patients with low expression of PTPLAD1. PTPLAD1 overexpression suppresses the metastasis of CRC *in vivo* and* in vitro* by inhibiting the Raf/ERK signaling-mediated EMT and mitofission. Mechanically, PTPLAD1 binds with PHB *via* its middle fragment (141-178 amino acids) and induces dephosphorylation of PHB-Y259 to disrupt the interaction of PHB-Raf, resulting in the inactivation of Raf/ERK signaling. Our results unveil a novel mechanism in which Raf/ERK signaling activated in metastatic CRC induces EMT and mitochondrial fission simultaneously, which can be suppressed by PTPLAD1. This finding may provide a new paradigm for developing more effective treatment strategies for CRC.

## Introduction

Cancer metastasis is a multi-step process resulting from signaling pathway disorder 1, 2, rendering the most significant handicap for cancer therapy and the leading cause of cancer-related deaths 3. As a critical progress accounting for cancer metastasis, epithelial-mesenchymal transition (EMT) is a reversible process in cancer cells by acquiring the mesenchymal phenotypes and decreasing the epithelial features 4. Accumulating evidence suggests that mitochondria are implicated in the regulation of cancer dissemination. It is well recognized that mitochondria are highly dynamic organelles that switch from fusion and fission status, aiming to meet the energy demand of cancer cells when they encounter diverse intercellular or extracellular stimuli 5. In human invasive breast carcinoma, dynamin-related protein 1 (Drp1) is significantly up-regulated in cancer tissue, promoting cancer metastasis through facilitating mitochondrial fission 6. Interestingly, we recently demonstrated that pharmacological induction of excessively asymmetric mitofission-associated cell death by switching the scission position from mitochondrial midzone to periphery represents a promising strategy for anticancer therapy 7. However, the upstream signaling/regulators of mitochondrial fusion-fission transition (MFT), as well as EMT in cancer remain unclear.

Raf/MEK/ERK1/2 signaling-mediated downstream gene networks are highly activated in transformed malignancies 8, key for cancer metastasis 9, 10, particularly in colorectal carcinoma (CRC). It is well known that c-Raf 1 initiates signaling cascades by phosphorylating MEK/ERK1/2 to activate downstream transcription factors (such as snail) to promote EMT 9, 11. In terms of mitochondrial dynamics, ERK2 was found to phosphorylate Drp1 at S616 and promote mitochondrial fission and tumor growth 12. We previously demonstrated that pharmacologically targeting ARF1/IQGAP1/ERK signaling inhibits Drp1-mediated mitochondrial fission, resulting in the suppression of CRC tumorigenesis 13. It seems that EMT and mitochondrial dynamics are implicated in Raf/MEK/ERK1/2 signaling, which is critical for cancer metastasis. The activation of Raf requires the involvement of Prohibitin 1 (PHB) located in plasma membrane lipid rafts 9. As a downstream of Ras or AKT, phosphorylated PHB (Y295) binds with Raf to initiate ERK cascades 14. Thus, inhibition of PHB phosphorylation represents an effective therapeutic strategy for cancer cell metastasis.

Protein tyrosine phosphatase-like A domain containing 1 (PTPLAD1) structurally includes a cs domain in the N-terminus, a linker peptide in the middle segment, and a protein tyrosine phosphatase-like (PTPLA) domain in the C-terminus 15, which supposes to exhibit phosphatase activity on tyrosine 16. PTPLAD1 was shown to interact with ELOVL protein and involved in long-chain fatty acid (VLCFA) synthesis 17. It was also reported in several diseases, such as HCV infection 18, 19, and type 2 diabetes disease 20. Nevertheless, its role in cancer cell metastasis remains obscure. In this study, we provided series of experimental evidence to show that PTPLAD1 is a novel anti-metastatic factor that suppresses both EMT and MFT by targeting PHB-Raf interaction to inactivate Raf/ERK/Snail signaling.

## Materials and Methods

### Cell lines and reagents

Human colon cancer cell lines SW480, SW620, HCT116, RKO, and normal small intestine epithelial cell FHs 74Int were purchased from ATCC (Rockville, MD), and the CRC cells were maintained in 1640 medium supplemented with 10% fetal bovine serum (FBS, Gibco, CA, USA) at 37°C with 5% CO_2_. HCT116 highly invasive cell line (HCT116-i8) was established using repeated invasion assays as previously described 21. All the cell lines were tested for mycoplasma contamination. U0126 and trichostatin A (TSA) were purchased from Selleck Chemicals (cat#S1102 and cat#S1045, Houston, TX, USA). U0126 (10 mM, 10 mg in 2.35 mL dimethyl sulfoxide (DMSO)), and TSA (10 mM, 5 mg in 1.65 mL DMSO) were respectively dissolved for *in vitro* usage.

### SILAC labeling, phospho-tyrosine enrichment LC-MS/MS, and bioinformatic analysis

SILAC labeling was performed as previously described 22. Briefly, HCT116 cells were maintained for 2 weeks in RPMI 1640 medium (cat# C11875500BT, ThermoFisher Scientific, Waltham, MA, USA) containing “heavy” amino acids (Arg-10 cat# CNLM-539-H-PK, Lys-8 cat# CNLM-291-H-PK; Cambridge Isotope Laboratories, Andover, MA) or “light” amino acids (Arg-0, Lys-0) supplemented with dialyzed FBS (ThermoFisher Scientific). The “heavy labeled” HCT116 cells were transfected with a siRNA against PTPLAD1 for 24 h, while “light labeled” HCT116 cells were transfected with the corresponding si-Control for 24 h. Lysates of both groups were collected and mixed in equal amounts. The mixed lysates were pre-washed with IgG (cat#sc-2025, Santa Cruz Biotechnology) and protein A/G Sepharose beads (cat#sc-2003, Invitrogen, Gaithersburg, MD) for 1 h at 4°C, and then incubated with the Phospho-Tyrosine Mouse mAb (cat#9411, Cell Signaling, Beverly, MA, USA) overnight at 4°C before being incubated with protein A/G Sepharose beads for 4 h. The beads were washed thrice with IP buffer (Beyotime, Jiangsu, China) and eluted in 2 × SDS/PAGE loading buffer (Beyotime) for SDS-PAGE separation. In-gel digestion was performed for three biological replicate experiments, and the peptides were analyzed by LTQ-Orbitrap mass spectrometer (ThermoFisher Scientific). Then, the raw data were analyzed through MaxQuant (Version 1.6.15, Max Planck Institute of Biochemistry). Carbamidomethylation (C) was specified as a fixed modification, while lysine acetylation, protein N-terminal acetylation, and oxidation (M) were specified as variable modifications. The results of peptide and protein were filtered by a 1% FDR. The enriched proteins were analyzed by Ingenuity Pathway Analysis (IPA, Ingenuity Systems, Redwood City, CA, USA).

### Plasmids, siRNAs, sgRNAs, and transfection

Flag-PTPLAD1, PTPLAD1-mCherry and Flag-PHB plasmids were generated by PCR amplification of sequences obtained from a colon cancer cDNA library and cloned into a pcDNA3.1 vector for transient transfection. These plasmids were subcloned to BamHⅠ or EcoRⅠ on lentiviral system (pLVX-CMV-puro) for establishing stable cell lines. The primers, siRNA sequences and sgRNA sequences used to generate plasmids are listed in Supplementary Tables 1 and 2. Transfections were performed using Lipofectamine^TM^ 3000 reagent (cat# L3000015, ThermoFisher Scientific) according to the manufacturer's recommendations. HCT116 cells stably expressing PTPLAD1, PHB, and PTPLAD1 plus PHB or GFP control genes were established with approximately 2 weeks of puromycin selection. The mutant constructs for pLVX-PHB^Y259A^ and pLVX-PHB^Y259D^ were created by the Mut Express II Fast Mutagenesis Kit V2 according to the manufacturer's manual (cat#C214-01/02, Vazyme Biotech Co., Ltd, Nanjing, China).

### Western blotting

The cell pellets were suspended in cell lysis buffer (cat#9803, Cell Signaling Technology) and incubated on ice for 30 min. The cell lysates were centrifuged at 14000 *g* for 30 min at 4°C, after which supernatant was mixed with loading buffer and boiled for 10 min at 95°C before being loaded onto a sodium dodecyl sulfate (SDS) polyacrylamide gel for electrophoresis. The proteins were subsequently transferred to polyvinylidene fluoride (PVDF) membranes (cat#1620177, Millipore, Billerica, MA, USA). After being blocked with 5% fat-free milk in Tris-buffered saline-Tween 20 (TBST), the membranes were probed with the appropriate primary antibodies, followed by the corresponding horseradish peroxidase (HRP)-conjugated secondary antibodies (cat#7074, 7076, Cell Signaling Technology). The signals were detected by Clarity Western ECL Substrate (cat#1705061, Bio-Rad, Hercules, CA, USA) and visualized by exposure to autoradiographic film. Antibodies against the following proteins were used for the experiment: PHB (cat#60092-1-Ig), ERK (cat#66192-1-Ig), and PTPLAD1 (cat#28572-1-AP), Raf1 (cat#51140-1-AP), Drp1 (cat#12957-1-AP), MFN1 (cat#13798-1-AP), MFN2 (cat#12186-1-AP), OPA1 (cat#27733-1-AP) and Fibronectin (cat#15613-1-AP) were obtained from Proteintech Group (Chicago, USA); Phospho-Drp1(Ser616, cat#4494), Vimentin (cat#5741), Snail (cat#3879), phospho-c-Raf (Ser338, cat#9427) was obtained from Cell Signaling Technology; E-Cadherin (cat#PTM-6222) was obtained from PTM BIO (Hangzhou, China); phospho-ERK1/2 (Phospho-ERK1-T202/Y204+ERK2-T185/Y187, cat#AP0472) was obtained from Ablconal (Wuhan, China).

### Boyden chamber invasion assay

Boyden chamber invasion assay was performed as previously described 23. In brief, cell invasion assays were performed with an 8 μm pore size invasion chamber coated with 5% Matrigel (cat#354234, BD Biosciences, San Jose, CA, USA) in serum-free 1640 medium. Cells were suspended in a serum-free medium and seeded into the upper chamber, and the lower compartment was filled with a complete medium. After incubation at 37°C and 5% CO_2_ for 36 h, the invaded cells adhering to the bottom surface of the chamber membrane were fixed with methanol and then stained with crystal violet (0.2% in methanol). Images of three different fields were captured from each membrane, and the number of invaded cells was counted.

### Quantitative real-time PCR (qRT-PCR)

qRT-PCR was performed as described previously 24. In brief, total RNA was isolated using TRIzol reagent according to the manufacturer's protocol (cat#15596018, Invitrogen). cDNA was synthesized using the PrimeScript™ II 1^st^ Strand cDNA Synthesis Kit (cat#6210A, Takara, Dalian, China). The mRNA expression levels of PTPLAD1 and the GAPDH control were detected by real-time PCR using SYBR® Premix Ex Taq™ II (cat#RR820A, Takara). The primers used in this study are listed in Supplementary Table 1.

### Co-immunoprecipitation (Co-IP)

The cell lysates were prewashed with IgG and protein A/G Sepharose beads for 1 h at 4°C, and the supernatants were incubated with the appropriate primary antibody overnight at 4°C before being incubated with protein A/G Sepharose beads for 4 h. The beads were washed thrice with lysis buffer and eluted in 2 × SDS/PAGE loading buffer for immunoblotting. For LC-MS/MS analysis, the lanes on the silver-stained gels were cut into several bands and digested in gel, after which LC-MS/MS analyzed the peptide mixtures.

### Confocal assay and Immunostaining

Cells with 60% confluence were transiently co-transfected with indicated plasmids or siRNA for 48 h and then fixed in 4% paraformaldehyde. The cells were subsequently stained with 2-(4-Amidinophenyl)-6-indolecarbamidine dihydrochloride (DAPI, cat#C1002, Beyotime Biotechnology, Shanghai, China) or mitoTracker-Red (cat#M7512, Thermo Fisher Scientific, Waltham, MA, USA) and observed by laser scanning confocal microscopy (Carl Zeiss AG, Jena, Germany). To determine mitochondrial morphology, cells were randomly selected for quantitative analysis and visually scored into three classifications (tubular, short tubular, and fragmented).

### Chromatin immunoprecipitation (ChIP) assay

The ChIP assay was performed by using the simple ChIP enzymatic chromatin IP kit (cat#9002, Cell Signaling Technology) according to the manufacturer's manual. Briefly, *in vivo* protein and DNA crosslinking was performed using 37% formaldehyde, followed by sonication and chromatin digestion. The protein-DNA complexes were immunoprecipitated by using the acetylated H3K9 antibody (cat#9649, Cell Signaling Technology) or negative control IgG antibody. After elution and reversal of crosslinking with proteinase K, the purified DNA was subjected to qRT-PCR analysis.

### Experimental metastasis assay in mice

Ethical consent was granted by the Ethical Committee Review Board of Jinan University. The Ethics Committee approved all animal experiments for Animal Experiments of Jinan University. Female NCG (NOD/ShiLtJGpt-Prkdc^em26Cd52^Il2rg^em26Cd22^/Gpt) mice (GemPharmatech, foshan, China) aged 5-8 weeks were maintained and cared under standard conditions according to the institutional guidelines for animal care. Cell lines with PTPLAD1 stable overexpression or knockdown and their corresponding negative control cells were constructed with HCT116 cells. For knockdown of PHB, the HCT116-PTPLAD1-KD cells was infected with lentiviral from pLKO-Tet-On-shPHB, and the knockdown of PHB was induced by doxycycline treatment. Approximately 1 × 10^6^ HCT116-luci cells were suspended in phosphate-buffered saline (PBS) and injected intravenously through the lateral tail vein of the mice. The injected mice were received U0126 or purina rodent chow with 2000 ppm doxycycline (C11300-2000i, research diets, USA) every two days. The metastatic activity was assessed by counting tumor nodes in the main organs (lung and kidney). The animals were euthanized at the end of the experiment (21 days), and the main organs were collected for further analyses, such as histological analysis.

### Tissue samples, tissue microarray, and immunohistochemistry (IHC)

Fifteen pairs of CRC tissues and adjacent normal tissues were used for qRT-PCR and ChIP assays. IHC was performed as described previously 25. Briefly, a human CRC tissue microarray (HColA180Su19) containing 94 cases of CRC tissues (Shanghai Outdo Biotech, Shanghai, China) with survival data was incubated with PTPLAD1 antibody (Proteintech). PTPLAD1 immunostaining was evaluated based on scores representing the percentage of positively stained tumor cells and the staining intensity grade. Two independent pathologists determined these scores. The percentages of positively stained tumor cells were scored according to the following scale: 1, < 25%; 2, 25-50%; 3, 51-74% and 4, 75-100%; A positive reaction was scored into 5 grades according to the intensity of staining: 1, 2, 3, 4 and 5. The product of the intensity and percentage scores was used as a final staining score. The score was considered as high (9) or low (< 9) and subjected to Kaplan-Meier analysis.

### Bioinformatics analysis

The expression of PTPLAD1 (HACD3) at different stages of TCGA-COAD was analyzed by using GEPIA (Gene Expression Profiling Interactive Analysis) webserver (http://gepia.cancer-pku.cn/). The DEGs of PTPLAD1 in TCGA-COAD (457 cases) and TCGA-READ (167 cases) were analyzed by Sangerbox (http://www.sangerbox.com/tool) 26. For the analysis of Mesenchymal/Epithelial ratio, the values of mesenchymal markers (VIM, CDH2, SNAI1/2 and MMP2/9) and epithelial markers (CDH1, TJP1/2) from TCGA-COAD or TCGA-READ patients were respectively summed to generate the ratio. In addition, the values of mitochondrial fusion markers (MFN1/2 and OPA1) and mitofission markers (FIS1 and DNM1L) were respectively summed to generate the mitochondrial Fusion/Fission Ratio.

### Statistical analysis

The results were analyzed by using GraphPad PRISM software (GraphPad Software Inc., San Diego, CA, USA), and the data from different experiments were compared by Student's t-test and expressed as the mean ± SD. Sample size in animal experiments was chosen based on the literature documentation of similar well-characterized experiments, and no statistical method was used to predetermine sample size. Pearson's chi-square test was performed to analyze the association between PTPLAD1 expression levels and categorical clinicopathological variables. Survival analysis was performed using the Kaplan-Meier method with the log-rank test. *p* < 0.05 was considered statistically significant.

## Results

### Loss of PTPLAD1 expression is associated with grim survival of CRC patients

**Figure 1:**
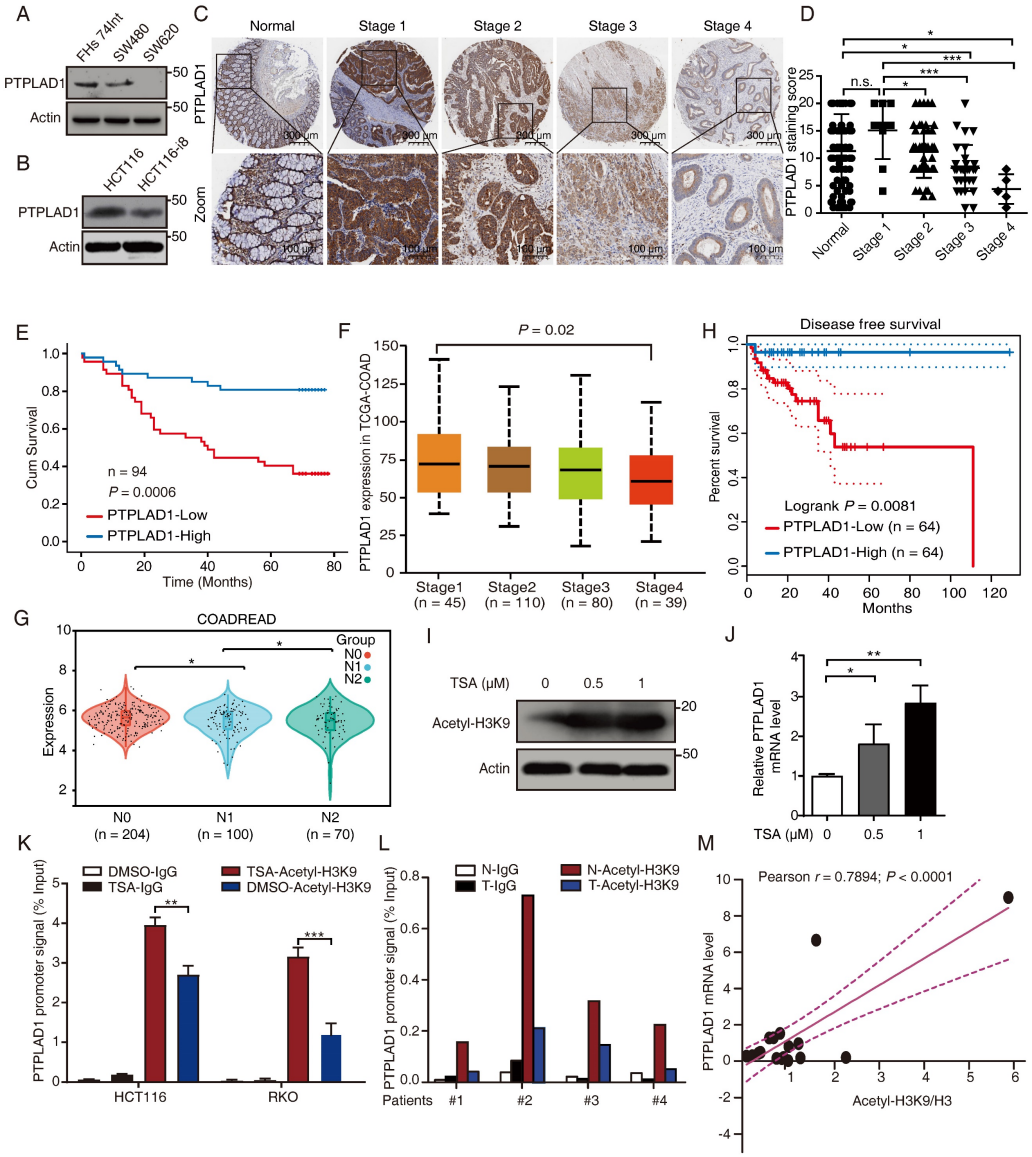
**PTPLAD1 is decrease in highly metastatic cancer cells and tissues and negatively associated with poor prognosis in CRC**. (A, B) The expression of PTPLAD1 in FHs74Int, SW480, SW620 (A), HCT116 and HCT116-i8 cells (B) was examined by western blotting assays. (C, D) The representative IHC images of PTPLAD1 staining in normal tissues and different stages (Stage 1-4) of CRC tissues (D). Bars, S.D.; n.s., no significant, *p < 0.05, ** p < 0.01, *** p < 0.001, compared with the staining score of stage 1 or normal as indicated (D). (E) Kaplan-Meier survival analysis of CRC patients according to the expression of PTPLAD1 by using median expression level as the cut-off point for survival analyses. High PTPLAD1 expression is significantly correlated with longer survival, and statistical significance was calculated by log-rank test (*p* = 0.0006). (F) The expression of PTPLAD1 was analyzed by using in TCGA-COAD, the expression level of PTPLAD1 was negatively correlated with individual cancer stages (tumor samples classified to stage1, stage2, stage3, and stage4). (G) The expression of PTPLAD1 in N0, N1 and N2 was analyzed by using in TCGA-COREAD. (H) Disease free survival analysis of TCGA-COAD patients according to the expression of PTPLAD1. (I, J) The effect of acetylation of histone H3K9 on the expression of PTPLAD1 in HCT116 cells. HCT116 cells were treated with DMSO or TSA (0.5 μM or 1 μM, 24 h), the acetylation status of histone H3K9 was determined by western blotting (I), and the transcription level of the *PTPLAD1* gene was evaluated by qRT-PCR (J). (K) HCT116 and RKO cells were treated with DMSO or TSA (1 μM, 24 h), ChIP assays were performed by using acetylated H3K9 antibody or IgG control, and the *PTPLAD1* promoter signal was detected by qRT-PCR. (L) ChIP assays were performed by using acetylated H3K9 antibodies in tumor tissues (T) and corresponding normal tissues (N), and the PTPLAD1 promoter signal was detected by qRT-PCR (n = 4). (M) The acetylation level of H3K9 and the expression level of PTPLAD1 in clinical tissues were detected by western blotting and qRT-PCR, respectively, and the correlation of H3K9 acetylation and PTPLAD1 expression was analyzed by correlation analysis (n = 15). Bars, S.D.; n.s., no significant, * p < 0.05, ** p < 0.01, as compared with the control group.

To determine the role of PTPLAD1 in several CRC lines with different invasive potentials, we performed western blot assay in SW480 and SW620 (two cell lines with different invasion abilities obtained from one patient), HCT116 and HCT116-i8 (HCT116 highly invasive cell screened by using 8 rounds of invasion assay) (**Supplementary Fig. 1A**), as well as FHs 74Int (normal small intestine epithelial cell). The result showed that the expression of PTPLAD1 was downregulated in highly invasive cells SW620 and HCT116-i8 (**Figs. 1A-B**). Next, we performed IHC assay to detect the level of PTPLAD1 in 94 cases of CRC tissues with survival and TNM data, and found that the expression level of PTPLAD1 did not significantly change between normal tissues and early stage of CRC tissues (stages 1 and 2), but it was gradually decreased in late stage of CRC tissues (stages 3 and 4). No significant difference was found between normal tissues and CRC tissues at Stage 1 (**Figs. 1C-D**). Besides, patients with low PTPLAD1 expression had shorter survival than those patients with high PTPLAD1 expression (*p* = 0.0006; **Fig. 1E**). Analysis of clinical pathological characteristics revealed that PTPLAD1 was negatively correlated with T-status (*p* = 0.0247), N-status (*p* = 0.0027), M-status (*p* = 0.0216) and late American Joint Committee on Cancer (AJCC) stages 3-4 (*p* = 0.0007) (**Table 1** and **Supplementary Fig. 1B**). The clinical significance of PTPLAD1 in CRC patients was further confirmed by using TCGA dataset. We found that PTPLAD1 expression was decreased along with tumor stage from stage 1 to stage 4 (*p* = 0.02;** Fig. 1F**) and from N0 to N2 (*p* < 0.05;** Fig. 1G**). Consistently, patients with the low level of PTPLAD1 displayed worse disease-free survival (**Fig. 1H**). These results suggest a tumor suppressive role of PTPLAD1 in CRC with clinical significance.

### Histone H3K9 acetylation promotes PTPLAD1 transcription

We then asked why the expression of PTPLAD1 is decreased during cancer development. A previous study reported that the PTPLAD1 gene is transcriptionally regulated by deacetylase inhibitors 27. Here, we found that trichostatin A (TSA), a selective histone deacetylase (HDAC) inhibitor, significantly enhanced the acetylation of histone H3K9 (**Fig. 1I**) and the mRNA level of PTPLAD1 (**Fig. 1J**) in HCT116. We detected the content of the promoter of PTPLAD1 with chromatin immunoprecipitation (ChIP) assay by using a histone H3K9 acetylation antibody, and found that the PTPLAD1 promoter was significantly accumulated at histone H3K9 when treated with TSA (**Fig. 1K**). We then collected 4 pairs of clinical CRC tissues and corresponding normal tissues and detected that the binding of H3K9 on PTPLAD1 promoter in normal tissues was stronger than that in tumor tissues (**Fig. 1L**). In addition, a positive correlation between H3K9 acetylation and PTPLAD1 transcription was found (*r* = 0.7894; *p* < 0.0001;** Fig. 1M**). These results indicate that the transcription of PTPLAD1 is positively regulated by H3K9 acetylation, and the low expression of PTPLAD1 in tumor tissues is attributed to the deacetylation of H3K9 on its promoter.

### Differentially expressed genes (DEGs) are regulated by PTPLAD1 in CRC

**Figure 2:**
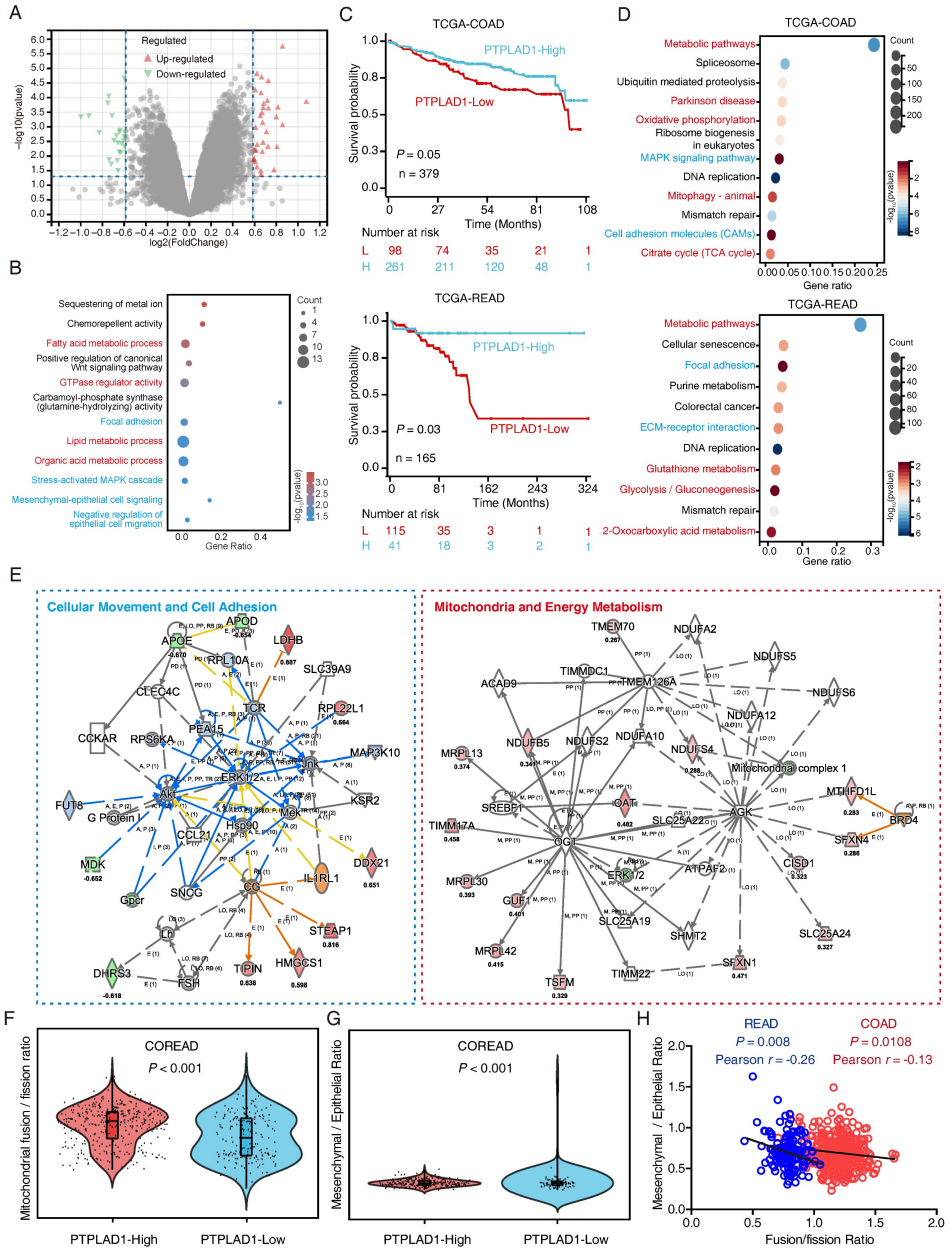
** Systematic analysis of the DEGs in CRC patients with PTPLAD1-high and PTPLAD1-low.** (A) The PTPLAD1-regulated differentially expressed genes (DEGs) in RKO cells were analyzed by RNA-Seq, the up-regulated genes (red) and down-regulated genes (green) were displayed by volcano plots. (B) KEGG enrichment analysis of the DEGs between RKO-PTPLAD1 and RKO-Ctrl. (C) Kaplan-Meier survival analysis of TCGA-COAD and TCGA-READ patients according to the expression of PTPLAD1 by using optimal cut-off point for survival analyses. High PTPLAD1 expression is significantly correlated with longer survival in both COAD and READ, and statistical significance was calculated by log-rank test. (D) KEGG enrichment analysis of the DEGs in both COAD and READ. (E) IPA analysis the networks of overlapped genes between COAD and READ. (F, G) The mitochondrial Fusion/Fission Ratio (F) and Mesenchymal/Epithelial Ratio (G) in dataset combined COAD and READ were compared. (H) Correlation analysis of mitochondrial Fusion/Fission Ratio and Mesenchymal/Epithelial Ratio in COAD (*p* = 0.0108, Pearson r = -0.13) and READ (*p* = 0.008, Pearson r = -0.26) respectively.

To understand the mechanism of PTPLAD1 in CRC, we performed RNA-Seq to compare the DEGs between RKO overexpressing PTPLAD1 and control, a total of 60 DEGs were identified (**Fig. 2A**). As shown in **Fig. 2B**, these PTPLAD1-regulated DEGs were mainly enriched in mitochondria-associated metabolic progresses (fatty acid and lipid metabolic progresses) and metastasis (focal adhesion and Mesenchymal-epithelial cell signaling). Moreover, we analyzed the COAD and READ datasets from TCGA by dividing the patients into two groups, PTPLAD1-high and PTPLAD1-low, according to the expression level of PTPLAD1. Both COAD and READ patients with PTPLAD1-low displayed worse overall survival than PTPLAD1-high (**Fig. 2C**). We systematically analyzed DEGs by comparing the patients with PTPLAD1-high and PTPLAD1-low in COAD and READ respectively. As shown in heatmaps of both COAD and READ, patients with PTPLAD1-high exhibited remarkable distinct expression pattern to PTPLAD1-low (**Supplementary Fig. 2**).

Consistently, the DEGs in COAD and READ were mainly enriched in mitochondria associated pathways (metabolic pathways, oxidative phosphorylation and mitophagy) and cell movement associated pathways (MAPK signaling, cell adhesion, focal adhesion and ECM-receptor interaction) (**Fig. 2D**). IPA analysis of the 1457 overlapped DEGs showed that cell movement and mitochondrial pathway are two leading networks enriched in both COAD and READ (**Fig. 2E**). Since EMT and MFT are important biological processes involved in cancer cell movement and cancer mitochondrial metabolism 12, 28, respectively, which inspired us to speculated a rational link between PTPLAD1 and EMT/MFT. To confirm this, we analyzed the mitochondrial Fusion/Fission ratio and Mesenchymal/Epithelial ratio in the dataset combined COAD and READ. Interestingly, mitochondrial fission status and mesenchymal status co-occurred in COREAD patients with PTPLAD1-low (**Figs. 2F-G**). Negative correlations between mitochondrial Fusion/Fission Ratio and Mesenchymal/Epithelial Ratio were found in both COAD (*p* = 0.0108, Pearson r = -0.13) and READ (*p* = 0.008, Pearson r = -0.26) (**Fig. 2H**). Collectively, EMT and MFT are two occurring events implicated in PTPLAD1-associated cancer progression.

### PTPLAD1 suppresses CRC cell metastasis by inhibiting EMT and mitochondrial fission

**Figure 3:**
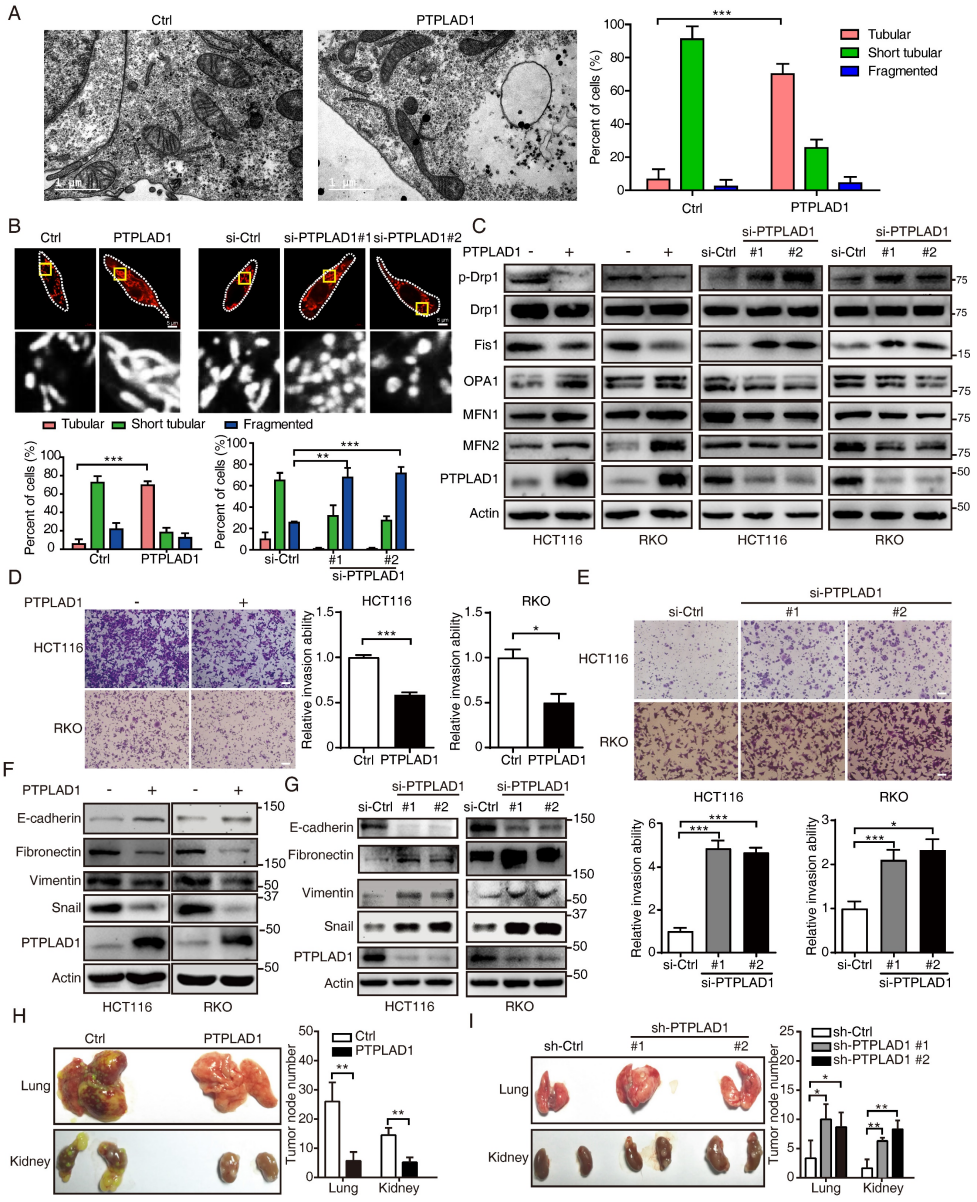
** PTPLAD1 suppresses CRC cell mitofission and metastasis*.*** (A) Electron micrographs of mitochondria in RKO cells transfected with empty vector or PTPLAD1-flag. Mitochondria in tubular, short tubular and fragment were statistically analyzed (n = 3). Scale bar, 1 μm in the left panels. (B) Mitochondrial morphology was visualized using MitoTracker probe in the indicated cells. Scale bars, 5 μm; (C) Expression levels of p-Drp1, Drp1, Fis1, OPA1, MFN1 and MFN2 in HCT116 and RKO cells with overexpression or knockdown of PTPLAD1 were determined by immunoblot. HCT116 and RKO cells were transfected with empty vector or PTPLAD1-flag, as well as with si-Ctrl or two siRNAs against PTPLAD1 (si-PTPLAD1-#1 or si-PTPLAD1-#2), and the cell lysates were subjected to immunoblotting analysis. (D, E) The effect of PTPLAD1 in CRC invasion. HCT116 and RKO cells were transfected with PTPLAD1-expressing plasmids (D) or siRNAs against PTPLAD1 (100 nM) (E), as well as the corresponding control vector or si-Ctrl. Then the invasion ability of these cells was evaluated by Boyden chamber invasion assays. Scale bar, 100 μm. (F, G) The expression of EMT markers in HCT116 and RKO cells transfected with PTPLAD1-expressing plasmids (F) or siRNAs (G) were determined by western blotting. (H, I) HCT116 cells with stable PTPLAD1 expression (H) or PTPLAD1 knockdown (I), as well as the corresponding control cells, were injected into mice intravenously. The mice were euthanized 4 weeks after injected, and the lung and kidney were isolated for counting the metastatic tumor nodes. Left, images of organs; right, statistics of tumor nodes in organs; 5 mice were used in each experimental group. Bars, S.D.; *p < 0.05, ** p < 0.01, *** p < 0.001 compared with the control group.

We next accordingly investigated the effect of PTPLAD1 on mitochondrial dynamics and EMT-mediated metastasis. By TEM analysis, we observed that mitochondria in PTPLAD1-overexpressing RKO cells displayed increased numbers of tubular mitochondria (**Fig. 3A**). We therefore validated the mitochondrial morphology using mitochondrial red fluorescent probe (MitoTracker probe) in CRC cells with overexpression or knockdown of PTPLAD1. As shown in **Fig. 3B**, mitochondria in RKO-PTPLAD1 displayed more elongated, and less short tubular and fragmented morphology, whereas knockdown of PTPLAD1 exhibited small sphere-like structures of mitochondria. Meanwhile, the expressions of phosphorylated Drp1 (p-Drp1, S616) and Fis1 were decreased, while OPA1, MFN1 and MFN2 were increased in HCT116 and RKO cells with PTPLAD1 overexpression, and a converse effect was found in CRC cells transfected with two siRNAs against PTPLAD1 (**Fig. 3C**), suggesting that PTPLAD1 negatively regulates mitochondrial fission in CRC. We next determined the effect of PTPLAD1 on CRC metastasis. As shown in **Figs. 3D-E**, overexpression of PTPLAD1 suppressed cell invasion, while knockdown of PTPLAD1 promoted the invasive ability of both CRC cell lines. Western blotting showed that PTPLAD1 overexpression increased the epithelial marker E-cadherin, and inhibited the mesenchymal markers fibronectin, vimentin and snail (**Fig. 3F**). Conversely, knockdown of PTPLAD1 decreased epithelial marker expression and promoted mesenchymal markers' expression (**Fig. 3G**), suggesting that PTPLAD1 suppresses CRC cell metastasis *via* inhibiting EMT process.

We further confirmed the suppressive effect of PTPLAD1 on cancer metastasis *in vivo*. HCT116 cell sublines with stable overexpression or knockdown of PTPLAD1 were generated and injected into NCG mice intravenously. Four to six weeks after the injection, the mice were euthanized, and the main organs (lung and kidney) were isolated for metastatic tumor node counting and pathological analysis. As shown in **Fig. 3H**, overexpression of PTPLAD1 significantly suppressed the multiple organ metastasis of CRC cells, while knockdown of PTPLAD1 led to more serious metastasis (**Fig. 3I**). Collectively, these results demonstrate that PTPLAD1 is a tumor suppressor that suppresses CRC metastasis *in vitro* and *in vivo* by inhibiting both EMT process and mitochondrial fission.

### PTPLAD1 suppresses the activation of Raf/ERK/Snail signaling pathway

**Figure 4:**
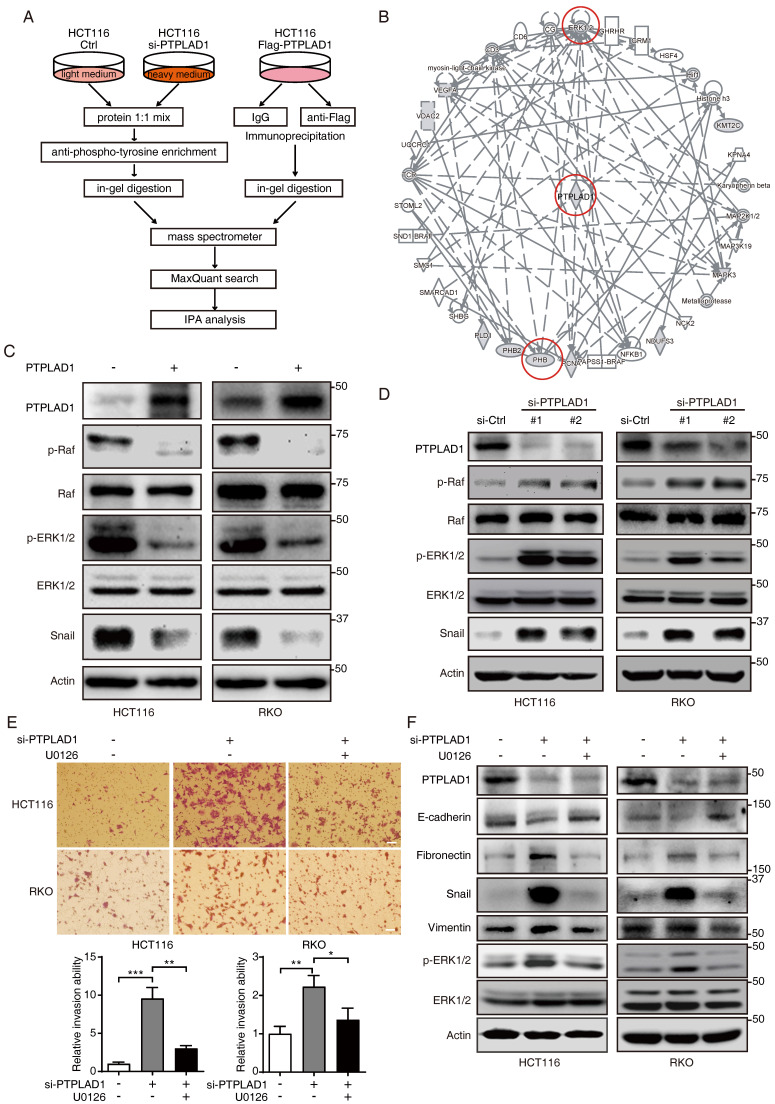
** PTPLAD1 suppresses the activation of the Raf/ERK/Snail signaling cascades.** (A) The workflow of IP-MS assay on determining PTPLAD1 binding partners/substrate. Anti-p-Tyr antibody was used for the enrichment of the lysates from HCT116 with (heavy chain) or without (light chain) PTPLAD1-KD. In addition, Co-IP assay against PTPLAD1-flag was performed by using a flag-tagged antibody, and the immunoprecipitated proteins were identified by mass spectrometry and ingenuity pathway analysis (B). (C, D) HCT116 and RKO cells were transfected with PTPLAD1-flag plasmids (C) or siRNAs against PTPLAD1 (D) and the corresponding controls. The expression of PTPLAD1, Raf, ERK1/2 and snail, as well as the p-Raf and p-ERK1/2, were determined by western blotting. (E, F) HCT116 and RKO cells were transfected with a siRNA against PTPLAD1 with or without the presence of U0126, the invasion ability of HCT116 and RKO cells was evaluated by Boyden chamber invasion assays (E). Scale bar, 100 μm; Histogram, statistics of invaded cells; Data are presented from three independent experiments; Bars, S.D.; * p < 0.05; ** p < 0.01; *** p < 0.001 compared with the indicated group; and the expressions of E-cadherin, fibronectin, snail, vimentin and p-ERK1/2 were detected by western blotting (F).

We next performed immunoprecipitation plus mass spectrometry (IP-MS) to investigate the signaling pathways and binding partners associated with PTPLAD1. Since PTPLAD1 has protein tyrosine phosphatase-like (PTPLA) domain in the C-terminus 15, we speculated that knockdown of PTPLAD1 will increase cellular p-Tyr level, anti-p-Tyr antibody was thus used for the enrichment of the lysate from HCT116 with or without PTPLAD1-KD. To improve the accuracy, Co-IP assay against PTPLAD1-flag was performed by using a flag-tagged antibody, and the immunoprecipitated proteins were analyzed by MS and IPA (**Fig. 4A; Supplementary Table 3**). The results showed that PTPLAD1-mediated protein-protein interaction (PPI) network was linked to ERK1/2 signaling cascades (**Fig. 4B**), consistent with our IPA result (**Fig. 2E**).

To confirm this, we transfected HCT116 and RKO cells with PTPLAD1-expressing plasmids, and found that the expressions of p-Raf, p-ERK1/2 and snail were significantly inhibited by PTPLAD1 (**Fig. 4C**). In contrast, silence of PTPLAD1 showed an opposite effect (**Fig. 4D**), indicating a negative regulation of PTPLAD1 on Raf/ERK/Snail signaling pathway. To further verify the role of PTPLAD1 in inactivating the ERK signaling pathway, HCT116 and RKO cells were treated with si-PTPLAD1 or the combination of si-PTPLAD1 and U0126 (a selective MEK/ERK pathway inhibitor) for 24 hours, we found that the invasion of HCT116 and RKO cells induced by PTPLAD1-knockdown was abrogated by U0126 (**Fig. 4E**). Likewise, the activation of both ERK, EMT, and MFT induced by PTPLAD1-knockdown was reversed by U0126 treatment (**Fig. 4E**; **Supplementary Fig. 3A**). These data indicate that PTPLAD1 suppresses CRC cell invasion by inactivating the Raf/ERK/Snail signaling pathway. Interestingly, employment of mitochondrial division inhibitor-1 (Mdivi-1) abolished the cell invasion promoted by PTPLAD1-knockdown (**Supplementary Fig. 3B**), suggesting that mitofission is required for PTPLAD1-deficiency mediated CRC metastasis.

### PTPLAD1 interacts with PHB *via* its middle fragment and C-terminus

**Figure 5:**
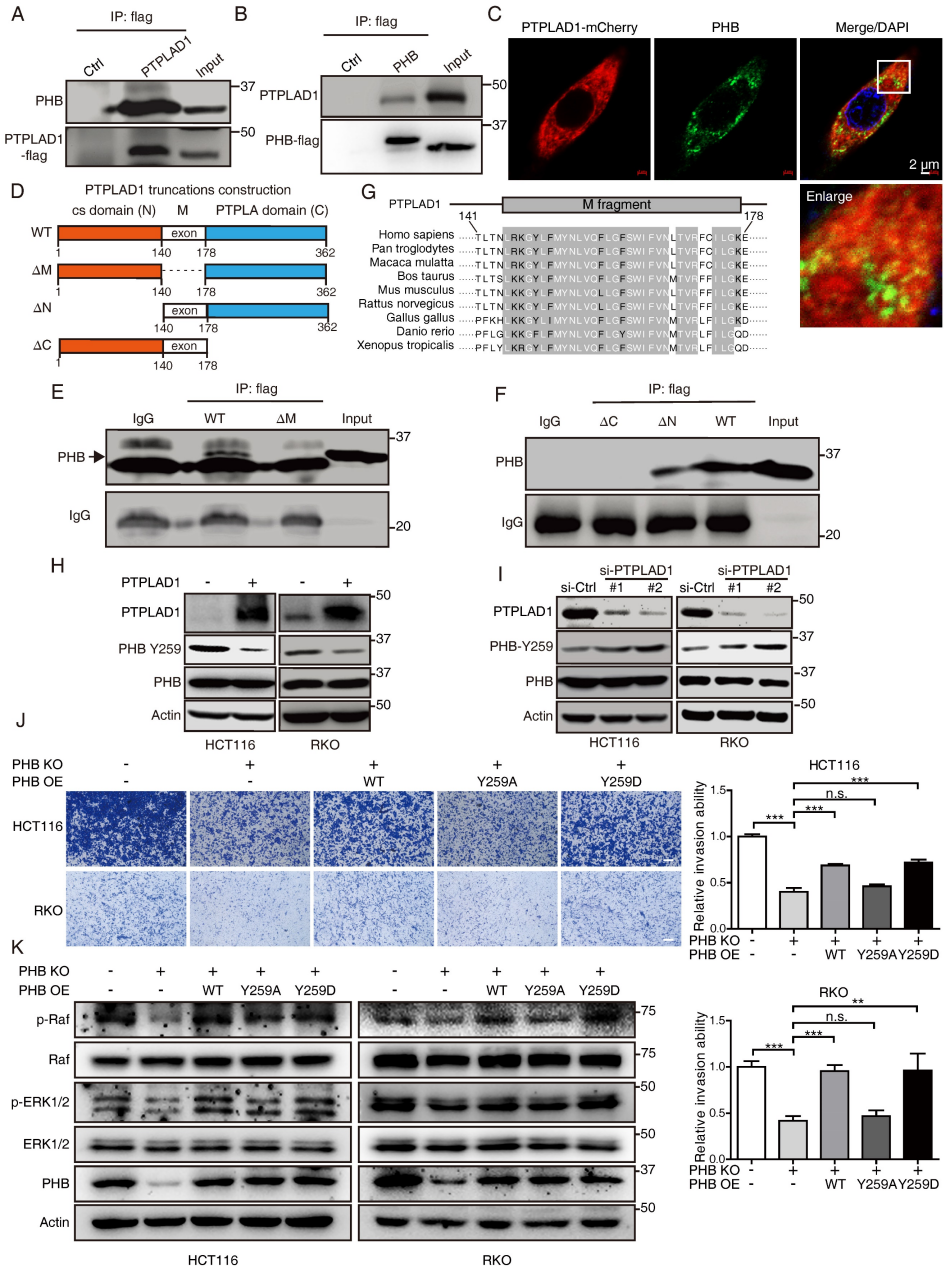
** PTPLAD1 interacts with PHB through the middle peptide and C-terminus.** (A, B) HCT116 cells were transfected with PTPLAD1-flag or PHB-flag plasmids, Co-IP assays were performed by using a flag antibody, and the expression of PHB (A) and PTPLAD1 (B) were detected by western blotting. (C) HCT116 cells were transfected with PTPLAD1-mCherry plasmids, and stained with PHB antibody, the subcellular localization of PTPLAD1 and PHB was performed by confocal analysis. PHB, green; PTPLAD1, red; DAPI was used to stain the nuclei. Scale bar, 2 μm. (D) Schematic diagrams of PTPLAD1 mutants including cs domain deletion(ΔN), M fragment deletion(ΔM) and PTPLA domain deletion (ΔC). (E) HCT116 cells were transfected with PTPLAD1-flag or ΔM-flag plasmids, Co-IP assays were performed by using a flag antibody, and the expression of PHB was detected by western blotting; IgG was considered as loading control. (F) HCT116 cells were transfected with ΔC, ΔM or PTPLAD1-flag (WT) plasmids individually, Co-IP assays were performed by using a flag antibody, and the expression of PHB was determined by western blotting. IgG was considered as loading control. (G) Alignment of the consensus M fragment sequence with amino acids 141-178 of human PTPLAD1 and the corresponding sequence from the indicated species. (H, I) HCT116 and RKO cells were transfected with PTPLAD1-expressing plasmids (H) or siRNAs against PTPLAD1 (I) and the corresponding vector controls, and the expression of PTPLAD1 and PHB, as well as PHB-Y259, were determined by western blotting. (J, K) PHB-deficient HCT116 and RKO cells were transiently transfected with PHB^wt^, PHB^Y259A^, PHB^Y259D^ plasmids respectively, and the invasion ability of these cells was evaluated by Boyden chamber invasion assays (J), Scale bar, 100 μm; the expression of PHB, Raf, ERK1/2, as well as the activation status of Raf and ERK1/2 were detected by western blotting (K). Bars, S.D.; * *p* < 0.05, ** *p* < 0.01, *** *p* < 0.001, ns, not significant.

Among the PTPLAD1 binding partners identified in **Fig. 4B**, PHB was reported as an important regulator in Raf/ERK signaling 9, and thus attracted our further investigation. HCT116 cells were transfected with PTPLAD1- or PHB-expressing plasmids, Co-IP assays using a flag-tagged antibody verified an interaction between PTPLAD1 and PHB (**Fig. 5A-B**). Confocal microscopy also showed a partial location overlap between PTPLAD1 and PHB in CRC cells, supporting the interaction between PTPLAD1 and PHB (**Fig. 5C**). We then sought to determine the interaction of PTPLAD1 and PHB in detail. The structure of PTPLAD1 includes cs domain (N-terminus, 1-140 aa), PTPLA domain (C-terminus, 178-362 aa) and middle fragment (38 aa) in between (M, 141-177aa). We thus constructed plasmids containing flag-tag fused with the cs domain (N), PTPLA domain (C) or middle fragment (M) and found that none of these three PTPLAD1-truncated mutants can bind with PHB, respectively (**Supplementary Fig. 4A**). Interestingly, PTPLAD1-truncated mutant with cs domain deletion (ΔN, 141-362 aa), but not PTPLA domain deletion (ΔC), showed strong interaction with PHB (**Figs. 5D**-**F**), indicating that the M fragment and C-terminal are both necessary for PTPLAD1 to bind with PHB. Sequence alignment of the M fragment is evolutionarily conserved throughout multiple species (**Fig. 5G**), suggesting a critical role of the M fragment in the molecular function of PTPLAD1, regarding the fact that M fragment deletion is insufficient for PTPLAD1-PHB interaction.

### PTPLAD1 inhibits Raf/ERK/Snail signaling by dephosphorylating PHB at Y259

We detected the effect of PTPLAD1 on PHB, and found that alteration of PTPLAD1 did not influence the protein level of PHB (**Figs. 5H-I**). PHB is essential for ERK signaling activation by interacting with Raf 9, 14, and the phosphorylation of Y259 on PHB is correlated with the invasion of cancer cell 14. We next examined the effect of PTPLAD1 on the phosphorylation of PHB. Notably, we found that PTPLAD1 significantly reduced the phosphorylation of PHB at Y259 (**Fig. 5H**), while knockdown of PTPLAD1 improved the phosphorylation level (**Fig. 5I**), suggesting that dephosphorylation of PHB-Y259 is regulated by PTPLAD1.

To confirm the role of p-Y259 of PHB on CRC metastasis, firstly, endogenous PHB-deficient cells were established by using CRISPR/Cas9 technology (**Supplementary Fig. 4B**), phosphorylation-mimetic Y259D mutant and dephosphorylation-mimetic Y259A mutant were then re-introduced into PHB-knockout (KO) cells, respectively. We found that knockout of PHB inhibited cancer cell invasion, while the reconstituted expression of wild type (WT) PHB or phosphorylation-mimicking PHB-Y259D mutant, but not PHB-Y259A, restored the invasive ability of CRC cells (**Fig. 5J**) and the activation of Raf and ERK (**Fig. 5K**). This observation indicates that phosphorylation of Y259 on PHB is required for Raf-ERK signaling-mediated cancer metastasis.

### PHB is required for PTPLAD1-mediated ERK signaling activation

**Figure 6:**
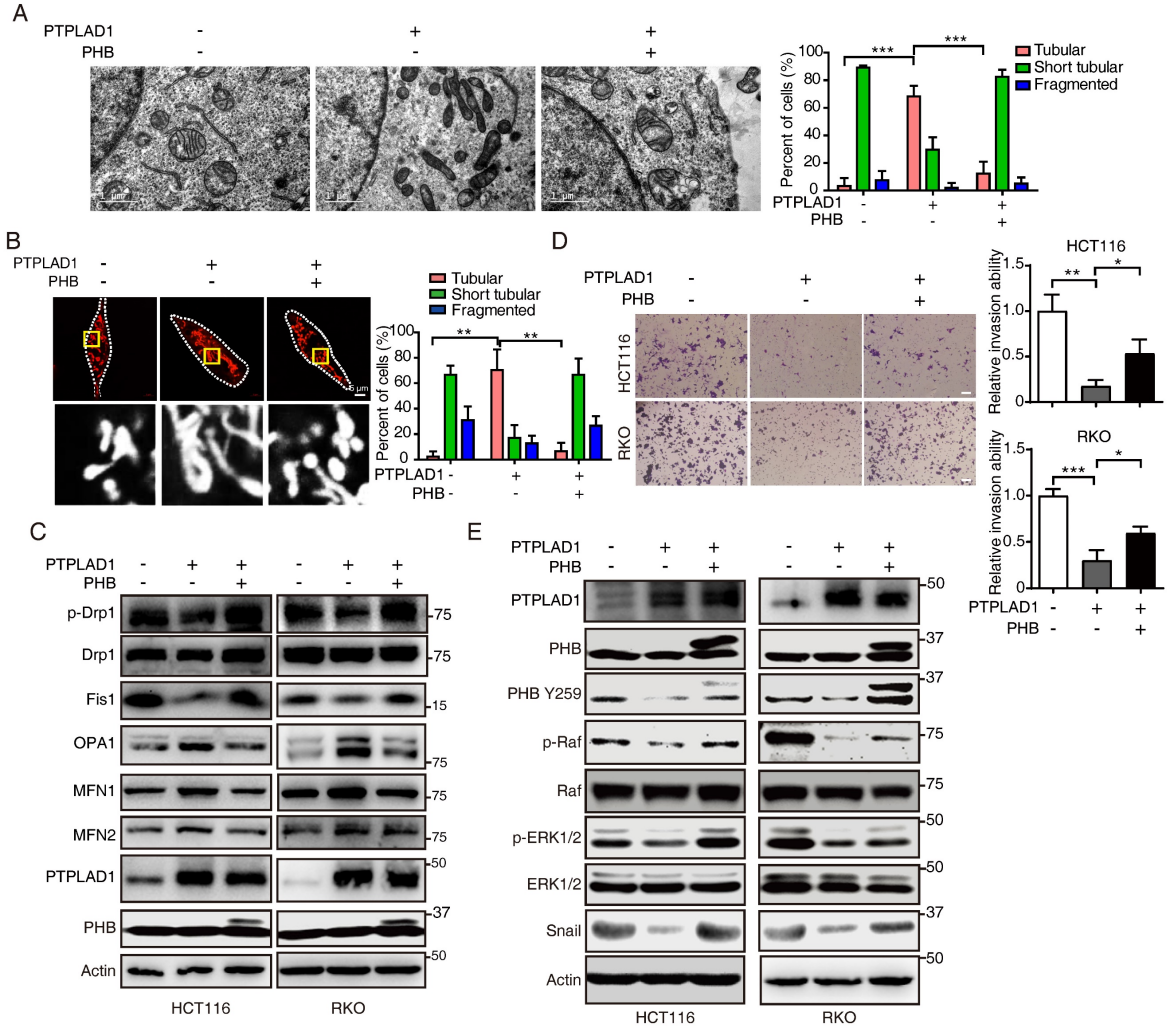
** PHB is required for PTPLAD1-mediated ERK signaling activation.** (A) Representative TEM images of RKO transfected with PTPLAD1 and/or PHB for 48 h, and the mitochondrial morphology with tubular and fragment were statistically analyzed (n = 3). Scale bars, 1 μm. (B) Mitochondrial morphology was visualized using MitoTracker probe in the indicated cells, and the mitochondrial morphology with tubular and fragment were statistically analyzed (n = 3) Scale bars, 5 μm. (C) Immunoblotting analysis of the expression levels of p-Drp1, Drp1, Fis1, OPA1, MFN1 and MFN2 in HCT116 and RKO cell with indicated transfection. (D, E) HCT116 and RKO cells were transfected with PTPLAD1-expressing plasmids alone or co-transfected with PHB-expressing plasmids, the invasion ability of cells was evaluated by Boyden chamber invasion assays (D), scale bar, 100 μm, and the expression of PTPLAD1, Raf, ERK1/2, PHB and Snail, as well as the activation status of Raf, ERK1/2 and PHB, were detected by western blotting (E). Bars, S.D.; * *p* < 0.05, ** *p* < 0.01, *** *p* < 0.001.

We next confirmed the roles of PHB and PTPLAD1 in mitochondrial dynamics and EMT. Induction of PHB changed the mitochondrial morphology in RKO cells expressing PTPLAD1 (RKO-PTPLAD1) from tubular to fragmented (**Fig. 6A**). Correspondingly, IF staining showed that the elongated mitochondria in RKO-PTPLAD1 cell were decreased after PHB overexpression (**Fig. 6B**), the rescued effect of PHB was further supported by the expression of mitofission markers (p-Drp1 and Fis1) and mitofusion markers (OPA1, MFN1 and MFN2) (**Fig. 6C**). In terms of cancer metastasis, we observed that the suppression of cell invasion induced by PTPLAD1 was recovered by PHB overexpression (**Fig. 6D**). Accordingly, the PTPLAD1-induced decrease of p-PHB, p-Raf, p-ERK1/2 and snail were restored by the expression of PHB (**Fig. 6E**).

**Figure 7:**
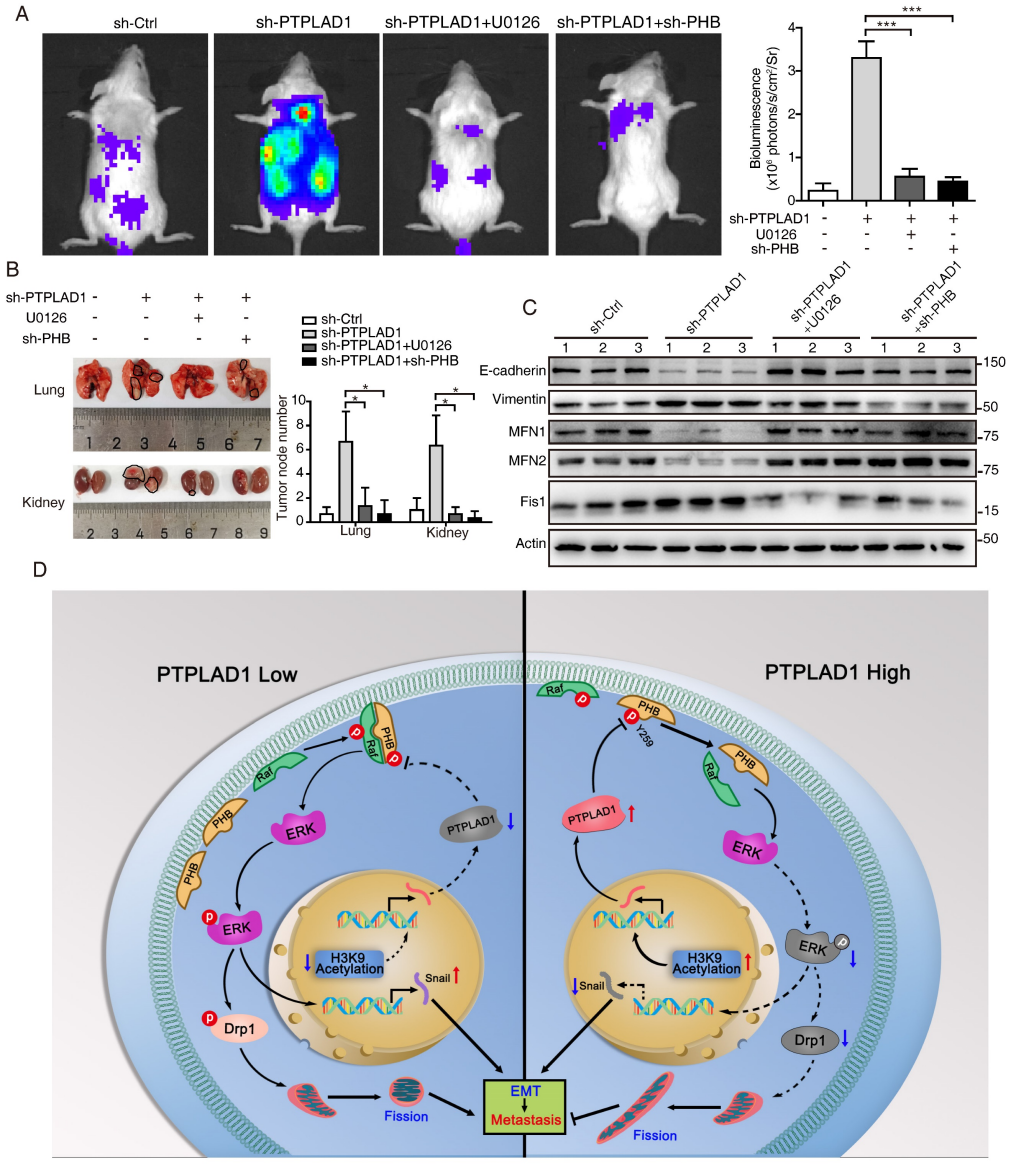
** PHB/Raf/ERK pathway-mediated EMT and MFT is required for PTPLAD1-regulated CRC metastasis.** (A-C) Knockdown of PHB or treatment of U0126 restored the pro-metastatic effect of PTPLAD1 deficiency. Bioluminescence imaging (A) and quantification of lung and kidney metastasis in NCG mice that were intravenously injected with HCT116-luci cells expressing sh-PTPLAD1, sh-PHB or sh-Ctrl, and indicated treatment. The lungs and kidneys were excised for imaging, and the metastatic nodules in lungs and kidneys were quantified (B). Bars, SD; n = 3; *p < 0.05; ***p < 0.001. Black circle, metastatic nodules. (C) The protein levels of EMT markers (E-cadherin and vimentin) and MFT markers (MFN1, MFN2 and Fis1) in lungs of above treated NCG mice were determined by western blotting. (D) Schematic diagram summarizing the role of PTPLAD1 in suppressing CRC metastasis. Briefly, PTPLAD1 is upregulated in low metastatic CRC by H3K9 acetylation, and the expression of PTPLAD1 represses the PHB/Raf/ERK signaling pathway by dephosphorylating PHB at Y259 and finally induces the suppression of epithelial-to-mesenchymal transition and mitochondrial fusion-to-fission transition.

We further determined the role of PHB/Raf/ERK pathway-mediated EMT and MFT in PTPLAD1-regulated CRC metastasis. We observed that knockdown of PHB restored the mitofission and cell invasion induced by PTPLAD1-KD (**Supplementary Figs. 5A-C**), while the activation of p-PHB, p-Raf, p-ERK1/2 and snail mediated by PTPLAD1-KD was abolished by the deletion of PHB (**Supplementary Fig. 5D**). In line with the results *in vitro*, in an NGC mice metastasis model, HCT116 cells expressing PTPLAD1-KD had increased metastatic foci forming ability in lung and kidney, while treatment of U0126 or knockdown of PHB displayed hampered metastatic foci formation (**Figs. 7A-B**). Moreover, we detected the protein levels of EMT markers (E-cadherin and vimentin) and MFT markers (MFN1/2 and Fis1) of the lung of mice, and found that E-cadherin and MFN1/2 decreased, vimentin and Fis1 enhanced by PTPLAD1-KD could be rescued by U0126 or PHB-KD (**Fig. 7C**). These *in vitro* and *in vivo* results suggest that PTPLAD1 suppresses mitochondrial fission and EMT of CRC by inactivating PHB/Raf/ERK signaling pathway. In addition, we collected 6 cases of CRC tissues, and found that 3 cases of CRC patients with PTPLAD1-low displayed high level of EMT (evidenced by the increase of vimentin and decrease of E-cadherin), mitofission (evidenced by the increase of Fis1 and decrease of MFN1/2) and ERK signaling activity (evidenced by the upregulation of p-ERK) (**Supplementary Fig. 5E**). These results provide strong clinical evidences to confirm the role of PTPLAD1-related PHB/Raf/ERK signaling in CRC metastasis.

In conclusion, the phosphorylation of PHB at Y259 is critical for PHB/Raf/ERK/Snail signaling, and the dephosphorylation of PHB-Y259 by PTPLAD1 leads to the inactivation of PHB-Raf and consequently EMT and mitochondrial fission.

## Discussion

Hyperactive Raf/ERK signaling is essential for the malignant transformation of CRC, leading to poor clinical outcomes. Increasing agents have been developed for targeted therapies against this signaling 29. However, satisfactory efficacy has not yet been achieved in clinic. This burden reflects an urgent need for deeper mining the diversity of molecular events implicated in the Raf/ERK signaling network to propel the development of effective therapeutic strategies to target cancer in novel ways. The current study discoveries the co-occurring of two critical biological progresses, EMT and MFT, upon the activation of Raf/ERK signaling. We identified PTPLAD1 as a novel metastatic suppressor for its function in inactivating Raf/ERK signaling, thus suppressing snail-mediated EMT and Drp1-mediated mitochondrial fission. Mechanically, PTPLAD1 binds with PHB *via* its middle fragment and decreases phos-Y259 of PHB to disrupt the interaction of PHB-Raf, consequently suppressing CRC metastasis. Transcriptionally regulated by histone H3K9 acetylation, PTPLAD1 frequently lost in late-stage CRC or high-invasive cells, rendering a high activity of the PHB-Raf complex (**Fig. 7D**).

Continuous scission of extended mitochondrial networks into smaller particles was recently reported to contribute to cancer metastasis in multiple cancers, such as breast cancer 30. This study revealed that EMT and MFT are two vital features characterizing the differences between CRC patients with PTPLAD1-high and -low. The co-occurrence of the two phenotypes driven by MAPK/ERK signaling indicates that the metastatic potential of cancer cells may be associated with mitochondrial dynamic status. Drp1 is a critical mitochondrial fission-related protein that translocates from cytoplasm to the outer mitochondrial membrane upon GTP hydrolysis to mediate mitochondrial fragmentation 31. The activity of Drp1 relies on its phosphorylation at serine 616 (S616) that is regulated by several kinases. ERK2 and CDK5 are the well-established upstream regulators of Drp1 that supports a link between mitochondrial fragmentation and tumorigenesis 12, 32. The nuclear translocation of ERK induced by Raf activation can promote the expression of a wide range of EMT-associated genes, such as snail 33, 34. By regulating Drp1, ERK serves as a signal hub, and snail can explain the crosstalk between mitochondrial dynamics and EMT.

PHB protein is a Raf activator that regulates diverse biological processes in cancer, including proliferation, differentiation and invasion 35, 36. Phosphorylation of PHB located in the cell membrane links to the activation of PI3K/AKT and Raf/ERK pathways 9. It has been reported that T258 and Y259 of PHB are respectively phosphorylated by AKT and insulin 37, and phosphorylation of T258 has been proved to directly activate Raf. Here our results certified that Y259 phosphorylation of PHB is also necessary for Raf activation, as evidenced by the fact that re-expression of PHB Y259D, rather than Y259A, can activate Raf/ERK signaling and sustain CRC mitofission and metastasis.

Given the importance of Y259 phosphorylation on PHB-Raf interaction, it is not hard to understand that Y259 dephosphorylation of PHB by PTPLAD1 results in the inactivation of Raf/ERK signaling, leading to the suppression of CRC metastasis. This study further demonstrated that the PTPLA domain (C) and middle fragment (M) of PTPLAD1 are required for the binding with PHB. Surprisingly, the middle fragment of PTPLAD1 plays an indispensable role in the binding with PHB, probably due to the structural requirement for the interaction. As for whether PTPLA domain of PTPLAD1 exhibits tyrosine phosphatase activity remains further investigation, at least, our study observed that PTPLAD1 negatively regulated PHB-Y259 phosphorylation, which was essential for ERK signaling mediated EMT and MFT.

PTPLAD1 was previously reported to be involved in long-chain fatty acid synthesis 17, cell apoptosis 38 and viral replication regulation 18, 19, its role in cancer metastasis remains unknown, however. This study for the first time revealed that PTPLAD1 plays a tumor-suppressive role through inactivating Raf/ERK signaling. Currently, Raf/MEK inhibitors like vemurafenib and cobimetinib still achieve unsatisfactory efficacies in treating CRC bearing BRAF mutation because of the negative feedback activation of EGFR 39. Thus, combination therapy consisting of BRAF and EGFR inhibitors displays synergistic anticancer effects on CRC. We here are dedicated to identifying the upstream regulators of Raf/ERK signaling that are functionally next to EGFR, demonstrating the clinical potential of PTPLAD1 in regulating PHB-Raf interaction to repress CRC metastasis. This work disclosed the precise functional modulation of PTPLAD1 in orchestrating EMT and MFT co-occurring in metastatic CRC, advancing our knowledge of CRC pathogenesis and allowing new possibility for developing novel therapeutic strategies against CRC.

## Supplementary Material

Supplementary figures and tables.

## Figures and Tables

**Table 1:** Clinical characteristics of CRC patients and the level of PTPLAD1 protein expression in tumor tissue specimens

Variables	Number (%)	PTPLAD1 protein expression			
		Low	High	*P*-value	
**Gender**				0.6799	
Male	48 (51.06)	25	23		
Female	46 (48.94)	22	24		
**Age**				0.5831	
<55 year	16 (17.02)	9	7		
≥55 years	78 (82.97)	38	40		
**T status**				**0.0247**	
T1-2	11 (11.7)	2	9		
T3-4	83 (88.30)	45	38		
**N status**				**0.0027**	
Negative	60 (63.83)	23	37		
Positive	34 (36.17)	24	10		
**M status**				**0.0216**	
Negative	89 (94.68)	42	47		
Positive	5 (5.31)	5	0		
**AJCC stage**				**0.0007**	
1-2	58 (61.7)	21	37		
3-4	36 (38.3)	26	10		
**Pathological grade**				0.3981	
Ⅰ-Ⅱ	79 (84.04)	38	41		
Ⅲ-Ⅳ	15 (15.96)	9	6		

*P*-value: Chi-Square Test JACC, American Joint Committee on Cancer version 7 staging Pathological grade: Ⅰ-Ⅱ, well/moderate differentiation; Ⅲ-Ⅳ, poor/undifferentiated
