# Supplementary material for: PTPLAD1 Regulates PHB-Raf Interaction to Orchestrate Epithelial-Mesenchymal and Mitofusion-Fission Transitions in Colorectal Cancer
Source: Int J Biol Sci. 2024 Mar 25;20(6):2202–18. doi: 10.7150/ijbs.82361 (PMC11008263; doi:10.7150/ijbs.82361)
Supplement: Supplementary file 1 — Supplementary figures and tables. [file ijbsv20p2202s1.pdf]

Supplementary Information for

PTPLAD1 Regulates PHB-Raf Interaction to Orchestrate Epithelial-Mesenchymal and Mitofusion-Fission Transitions in Colorectal Cancer

Running Title: Antimetastatic Role of PTPLAD1 in Colorectal Cancer

Supplementary Table 1: Primers

Name	Primer (5'-3')
PTPLAD1-F	CGCGGATCCATGGAGAATCAGGTGTTGACGCCGC
PTPLAD1-R	CGCGAATTCTTACTTGTCATCGTCGTCCTTGTAGTC GTGGATCTTTTTCTTTTTTTGTC
PHB-F	CGCGGATCCCATGGCTGCCAAAGTGTTT
PHB-R	CGCGAATTCTTACTTGTCATCGTCGTCCTTGTAGTC CTGGGGCAGCTGGAGGAGCACGGAC
PTPLAD1-qPCR-F	GCCTAAATAAACGCCGACT
PTPLAD1-qPCR-R	AGAATCGCACAGTCAGGTT
GAPDH-qPCR-F	GAAGGTGAAGGTCGGAGTC
GAPDH-qPCR-R	AAGATGGTGATGGGATTTC
PTPLAD1-N-F	CTCGGATCCATGGAGAATCAGGTGTTGACGC
PTPLAD1-N-R	CGCGAATTCTTACTTGTCATCGTCGTCCTTGTAGTC TTCAGGAGAGCCTTCGCTTTCC
PTPLAD1-C-F	CGCGGATCCTCCTTTTATGACACATTCCATAC
PTPLAD1-C-R	CGCGAATTCTTACTTGTCATCGTCGTCCTTGTAGTC GTGGATCTTTTTCTTTTTTTGTC
PTPLAD1-M-F	CGCGGATCCATGAAGCAGGAAAAGCGACCAC
PTPLAD1-M-R	CGCGAATTCTTACTTGTCATCGTCGTCCTTGTAGTC GCAGAAATACATCATGTCAG
PTPLAD1-mCherry-F	CTCGGATCCATGGAGAATCAGGTGTTGACGC
PTPLAD1-mCherry-R	CGCGAATTCGTGGATCTTTTTCTTTTTTTGTC
PHB-GFP-F	CCGGAATTCACATGGCTGCCAAAGTGTTT
PHB-GFP-R	CGCGGATCCTTCTGGGGTGGGAGC
PHB Y259A-F	AACATCACCgcCCTGCCAGCGGGGCAGTCCGTG
PHB Y259A-R	TGGCAGGgcGGTGATGTTCCGAGAGCGTGAGAG
PHB Y259D-F	TCTCGGAACATCACCgACCTGCCAGCGGGGCAGT
PHB Y259D-R	TcGGTGATGTTCCGAGAGCGTGAGAGCTGGTA

F, forward; R, revese

Supplementary Table 2: siRNA, shRNA and sgRNA sequences

Name	Target sequence
si-Control	UUCUCCGAACGUGUCACGUTT
si-PTPLAD1 #1	GGAGAGACUCACAAAGCAGTT
si-PTPLAD1 #2	GUCCAUUCCAAUAUUCAAUTT
si-PHB	AGAGCUGGUACGCGAUGUCCUCUGC
sgPHB #1	CCACAATGTCCTGCACTCCA
sgPHB #2	AACTGAGATCCTCAAGTCAG
sh-PHB	AGAGCUGGUACGCGAUGUCCUCUGC

Supplementary Table 3: Proteins interacted with PTPLAD1

Protein ID	Protein Name	Protein Description
Q14240	IF4A2	Eukaryotic initiation factor 4A-II
Q9H936	GHC1	Mitochondrial glutamate carrier 1
P08574	CY1	Cytochrome c1, heme protein, mitochondrial
Q96HS1	PGAM5	Serine/threonine-protein phosphatase PGAM5, mitochondrial
P35232	PHB	Prohibitin
O00743	PPP6	Serine/threonine-protein phosphatase 6 catalytic subunit
P16402	H13	Histone H1.3
B3KVR1	B3KVR1	Sm domain-containing protein
Q8TAA3	PSMA8	Proteasome subunit alpha-type 8
J3QQ67	RPL18	60S ribosomal protein L18
P63244	GBLP	Receptor of activated protein C kinase 1
P12004	PCNA	Proliferating cell nuclear antigen
P12236	ADT3	ADP/ATP translocase 3
P27635	RPL10	60S ribosomal protein L10
J3KPX7	PHB2	Prohibitin
P62753	RS6	40S ribosomal protein S6
P45880	VDAC2	Voltage-dependent anion-selective channel protein 2
P21796	VDAC1	Voltage-dependent anion-selective channel protein 1
P11021	GRP78	Endoplasmic reticulum chaperone BiP
P62701	RS4X	40S ribosomal protein S4, X isoform
P50914	RL14	60S ribosomal protein L14
P15880	RPS2	40S ribosomal protein S2
P51148	RAB5C	Ras-related protein Rab-5C
P62258	1433E	14-3-3 protein epsilon
P25789	PSA4	Proteasome subunit alpha type-4
P26373	RL13	60S ribosomal protein L13
P36542	ATPG	ATP synthase subunit gamma, mitochondrial
H0Y368	H0Y368	Dolichol-phosphate mannosyltransferase subunit 1
P62829	RL23	60S ribosomal protein L23
O75489	NDUS3	NADH dehydrogenase [ubiquinone] iron-sulfur protein 3, mitochondrial
P05141	ADT2	ADP/ATP translocase 2
P62241	RS8	40S ribosomal protein S8

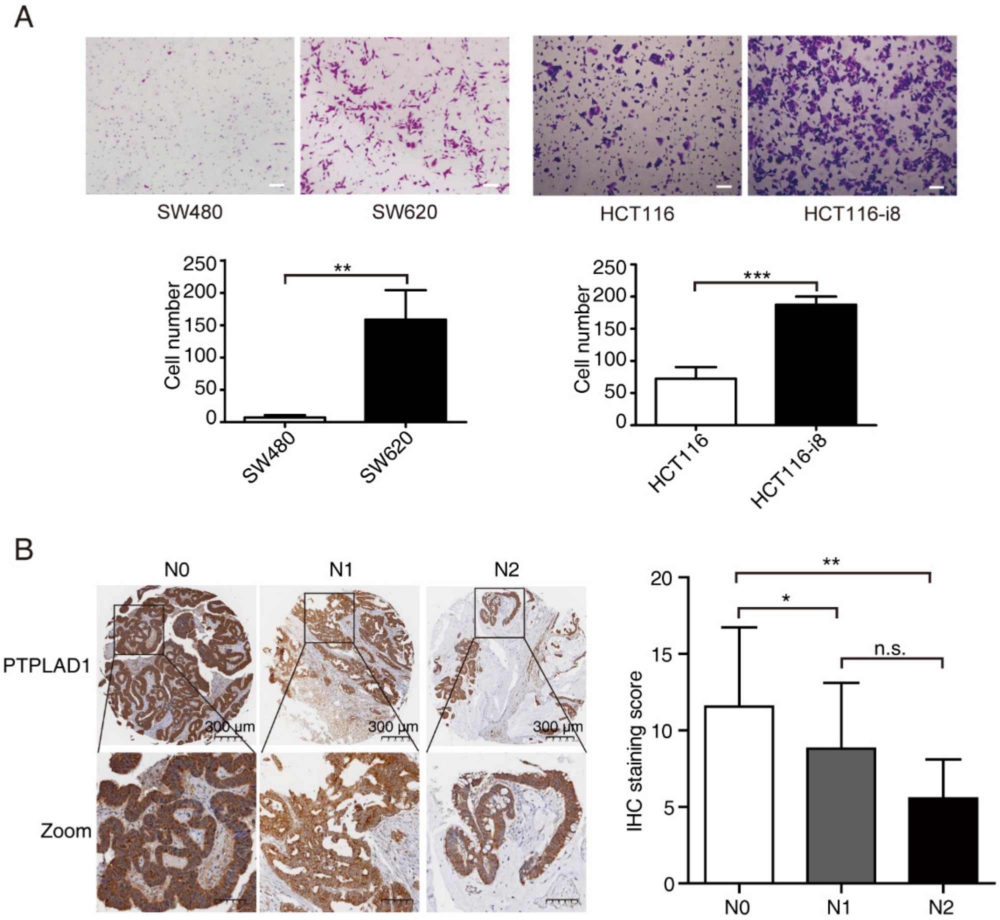

Supplementary Figure 1. (A) Comparison of the invasive abilities of SW480 and SW620, HCT116 and HCT116-i8. Upper, representative images of cell invasion; Lower, statistics of the invaded cells. Scale bar, 100 μm. (B) The representative IHC images of PTPLAD1 staining in N0, N1 and N2 stages of CRC tissues. Data are represented as the means ± SD; **p* < 0.05, ** *p* < 0.01; *** *p* < 0.001; n.s., no significance.

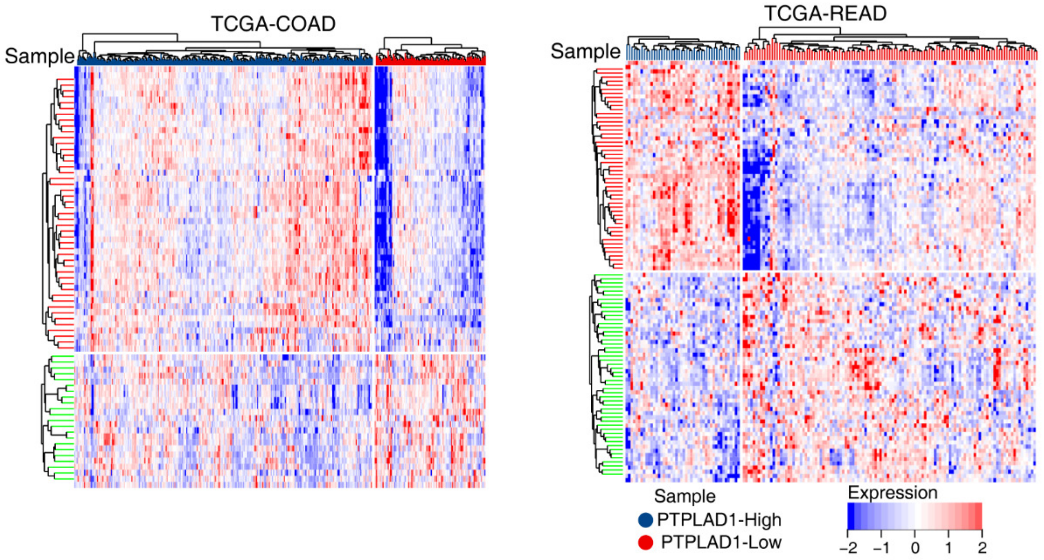

Supplementary Figure 2. Heatmap analysis showing the DEGs between PTPLAD1-high and PTPLAD1-low in both COAD and READ.

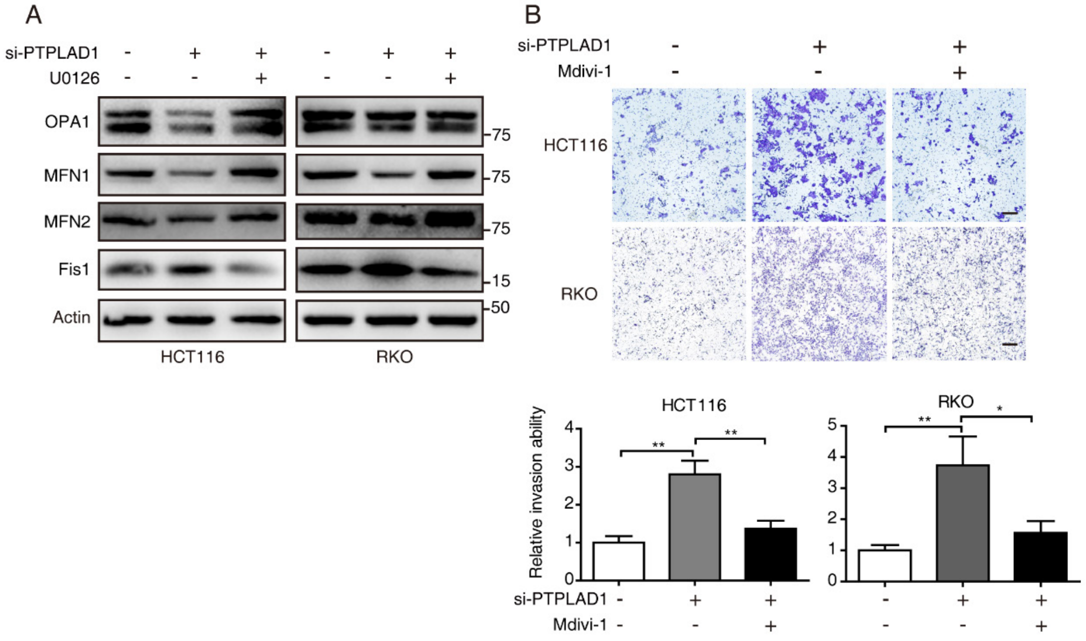

Supplementary Figure 3. (A) The expression of mitochondrial dynamics markers OPA1, MFN1, MFN2 and Fis1 in HCT116 and RKO cells transfected with a siRNA against PTPLAD1 and/or treated with U0126 were detected by western blotting. (B) The invasion ability of HCT116 and RKO cells treated with si-PTPLAD1 and/or Mdivi-1 was determined by Boyden chamber invasion assays. Scale bar, 200 µm. Bars, S.D.; * $p < 0.05$; ** $p < 0.01$ compared with the control group.

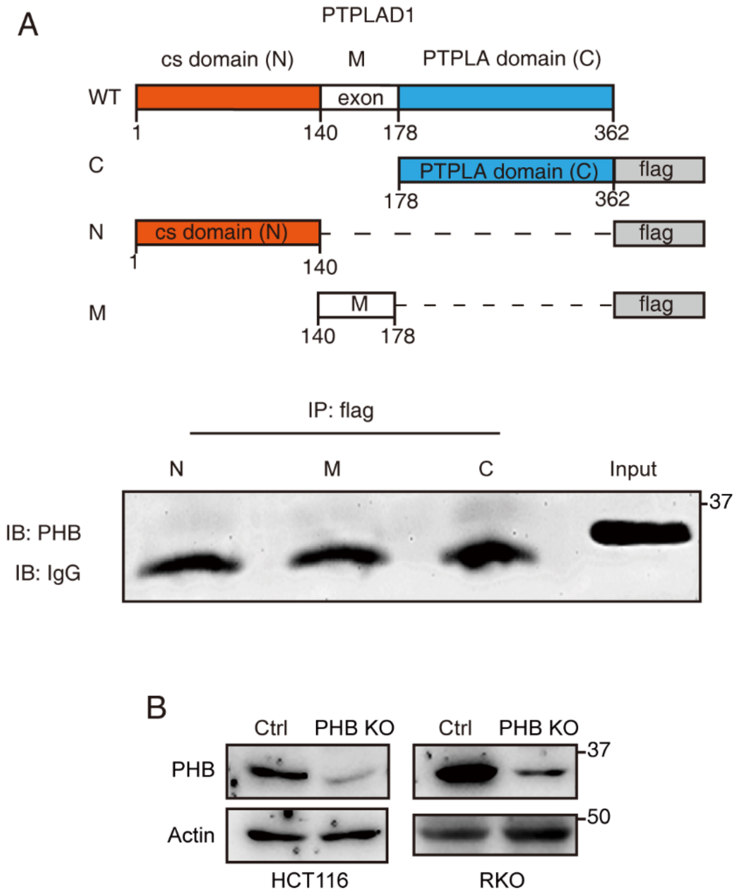

Supplementary Figure 4. (A) The individual N/M/C segment of PTPLAD1 could not interact with PHB. HCT116 cells were transfected with N-flag or M-flag or C-flag plasmid, and Co-IP assays were performed by using a flag antibody, and the expression of PHB was detected by western blotting. Up, structures of N/M/C segment genes; IgG was considered as the loading control. (B) PHB-deficient CRC cell lines were established by CRISPR/Cas9 technology.

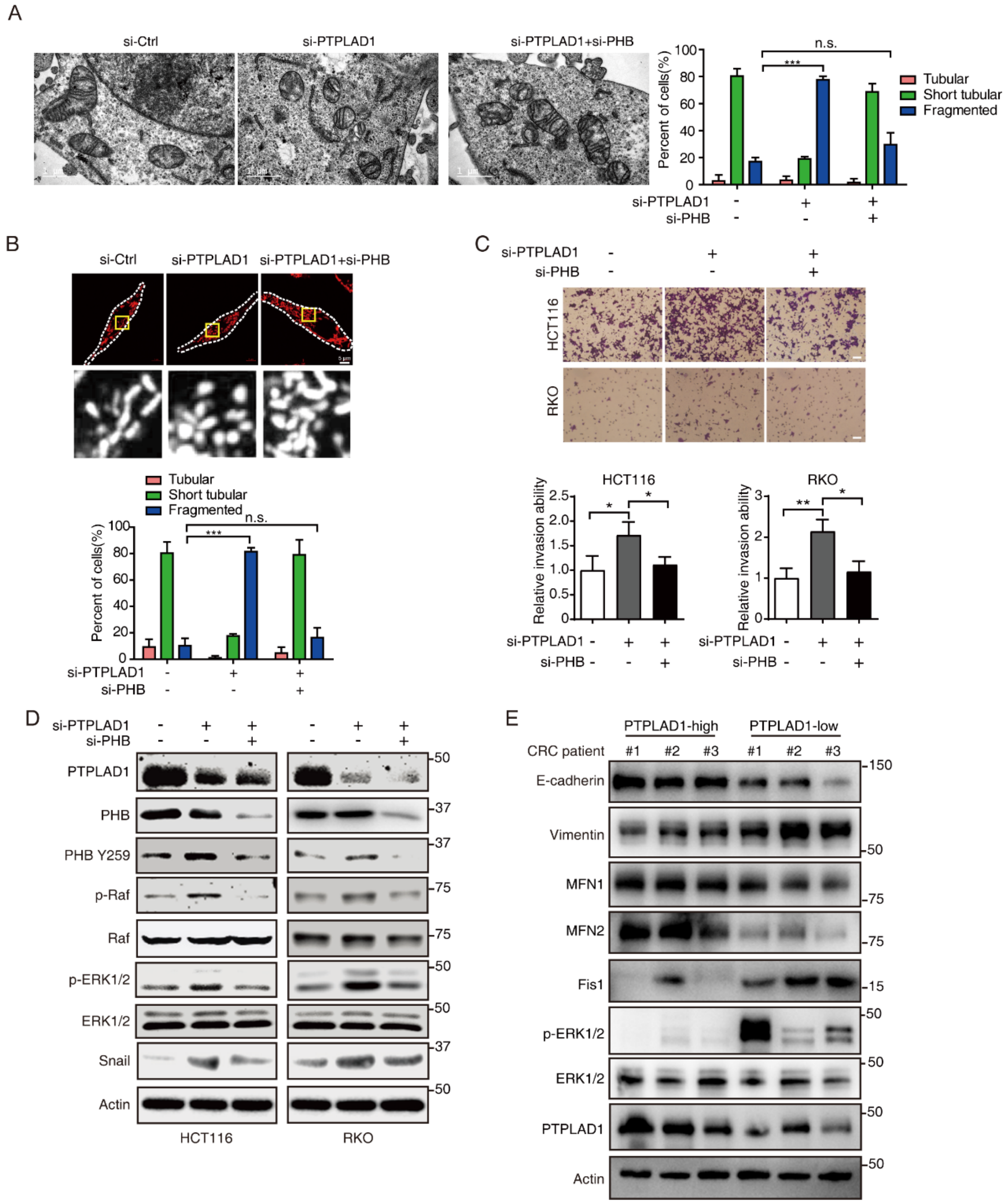

Supplementary Figure 5. (A) Representative TEM images of RKO transfected with si-PTPLAD1 and/or si-PHB, and the mitochondrial morphology with tubular and fragment were statistically analyzed (n = 3). Scale bars, 1 μm. (B) Mitochondrial morphology was visualized using MitoTracker probe in the indicated cells, and the mitochondrial morphology with tubular and fragment were statistically analyzed (n = 3) Scale bars, 5 μm. (C, D) HCT116 and RKO cells were transfected with a siRNA against PTPLAD1 or co-transfected with a siRNA against PHB, and the invasion ability of these cells was evaluated by Boyden chamber invasion assays (C). Scale bar, 100

µm; Histogram, statistics of the invaded cells. The expression of PTPLAD1, PHB, p-Y259-PHB, p-Raf, Raf, p-ERK1/2, ERK1/2 and Snail were detected by western blotting (D). Data are presented from three independent experiments. (E) The expressions of indicated EMT markers (E-cadherin and Vimentin), mitochondrial dynamics markers (MFN1, MFN2 and Fis1) and p-ERK1/2 in 6 cases of CRC patients were determined by western blotting. Data are represented as the means ± SD; *$p < 0.05$, ** $p < 0.01$; *** $p < 0.001$; n.s., no significance.
